# Toward High Performance 2D/2D Hybrid Photocatalyst by Electrostatic Assembly of Rationally Modified Carbon Nitride on Reduced Graphene Oxide

**DOI:** 10.1038/srep37318

**Published:** 2016-11-17

**Authors:** Jian Chen, Xiaochan Xu, Tao Li, Kannusamy Pandiselvi, Jingyu Wang

**Affiliations:** 1 Key Laboratory of Material Chemistry for Energy Conversion and Storage (Ministry of Education), School of Chemistry and Chemical Engineering, Huazhong University of Science and Technology, Wuhan, 430074, China

## Abstract

Efficient metal-free visible photocatalysts with high stability are highly desired for sufficient utilization of solar energy. In this work, the popular carbon nitride (CN) photocatalyst is rationally modified by acid exfoliation of molecular grafted CN, achieving improved visible-light utilization and charge carriers mobility. Moreover, the modification process tuned the surface electrical property of CN, which enabled it to be readily coupled with the oppositely charged graphene oxide during the following photo-assisted electrostatic assembly. Detailed characterizations indicate the formation of well-contacted 2D/2D heterostructure with strong interfacial interaction between the modified CN nanosheets (CNX-NSs) and reduced graphene oxide (RGO). The optimized hybrid (with a RGO ratio of 20%) exhibits the best photocatalytic performance toward MB degradation, which is almost 12.5 and 7.0 times of CN under full spectrum and visible-light irradiation, respectively. In addition, the hybrid exhibits high stability after five successive cycles with no obvious change in efficiency. Unlike pure CNX-NSs, the dye decomposition mostly depends on the H_2_O_2_ generation by a two-electron process due to the electron reservoir property of RGO. Thus the enhancement in photocatalytic activity could be ascribed to the improved light utilization and increased charge transfer ability across the interface of CNX-NSs/RGO heterostructure.

Efficient semiconductor photocatalytic processes have great potential in tackling some of social serious challenges, especially in solving environmental pollution and clean energy demand^1^,^2^. Among various semiconductor photocatalysts, graphitic carbon nitride (CN) with a comparable π-conjugated graphite structure has attracted considerable attention owing to its favorable visible light harvesting capability, tunable electronic structure, high chemical and thermal stability, abundant and low-cost precursors, and environmental friendliness^3^. Nevertheless, the visible light photocatalytic efficiency of bulk CN is quite low because of its limited sunlight absorption (below 460 nm)^4^. Furthermore, the weak van der Waals interaction between layers slows down the electrons transfer that leads to high recombination rate of photogenerated electron-hole pairs and low electrical conductivity^5^.

To overcome these problems, many attempts have been made to reduce the recombination losses and extend the light responsive region of CN catalysts. For example, several strategies such as fabricating porous structures and low-dimension architectures have been explored to improve the charge transportation channels or efficiency^6^,^7^,^8^. Because of a similar layered structure to graphite, bulk CN materials have recently been successfully exfoliated into graphene-analogue CN nanosheets (CN-NSs) with few atomic layers^9^,^10^,^11^,^12^. The resulting graphene-analogue structure brings about the increased mobility of charge carriers owing to the notable merits of two-dimension (2D) materials^12^. Moreover, the liquid-phase exfoliation process makes CN catalysts more water-dispersible, which is favorable for assembling on support materials when constructing heterostructures^13^. Unfortunately, such improvement is achieved with the sacrifice of the utilizable visible light energy. In comparison to bulk CN, the exfoliated CN-NSs exhibit a remarkable blue shift of the absorption edge to about 425 nm, presumably due to the decrease in conjugation length and the strong quantum confinement effect^9^,^10^,^11^,^12^. Hence the application of ultrathin CN-NSs as photocatalysts has been intrinsically restricted by the limited response within visible region. Recently, molecular grafting or chemical doping is found to be an effective method for tuning the electronic structure of CN and thus realizes the sufficient visible light absorption^14^,^15^,^16^,^17^. It has been reported that the CN frameworks will be modified when copolymerizing urea or dicyandiamide precursor with other organic monomers, such as aminobenzonitrile, barbituric acid, 2-aminothiophene-3-carbonitrile, *et al*. These co-monomers strongly altered the electronic structure of CN by extending π-conjugation system and creating surface molecular heterojunction. As a result, the molecularly grafted CN materials possess improving visible light absorption and charge separation efficiency^14^,^15^. In light of our previous work, exfoliating the molecularly grafted CN materials to 2D nanosheets will not severely hamper the visible light absorption though the blue shift always exists^18^. Therefore, the deficiency in optical response that caused by exfoliation could be circumvented by molecular grafting. This strategy provides the possibility for exfoliated sheet-like CN photocatalysts to utilize larger proportion of visible light.

In comparison to single-component photocatalyst, the recombination losses can be dramatically reduced by coupling with other materials to construct CN-based composites. There are many literatures about preparing composites with inorganic semiconductors such as TiO_2_, ZnO, WO_3_, BiVO_4_, *et al*.^19^,^20^,^21^,^22^. The transport efficiency of photogenerated charge carriers will be enhanced at the interface of heterojunction owing to the matched band structure. Generally, these composites comprise metal-containing semiconductors as the main component, which is unfavorable for economic benefits from practical application. In this regard, it is highly desirable to explore metal-free photocatalysts that function efficiently under visible light. Graphene, a typical 2D planar structure with very large π-conjugated system, appears to be a competitive candidate for coupling with CN because of its ultrahigh electron mobility (≈200000 cm^2^V^−1^s^−1^). The photogenerated electrons could rapidly transfer from the conduction band (CB) of CN polymer to graphene at their interface. The recombination of charge carries could therefore be greatly suppressed in the composite system^23^. Moreover, the analogous sp^2^-bonded carbon framework endows CN and graphene the compatible materials to form heterostructures^24^. Liao *et al*. fabricated graphene oxide modified g-C_3_N_4_ (GO/g-C_3_N_4_) with efficient photocatalytic capability under visible light irradiation by sonochemical approach^25^. Oh *et al*. reported a novel g-C_3_N_4_/reduced graphene oxide (g-C_3_N_4_/RGO) hybrid with good photocatalytic performance using a reflux method^26^. Another typical approach is *in situ* immobilization of g-C_3_N_4_ onto the graphene supports to the formation of g-C_3_N_4_/RGO composites by calcination of a mixture of CN precursor and GO at 550 °C in an inert environment^24^,^27^. Actually, the sublimation amount of CN precursor such as melamine or cyanamide is difficult to be controlled during thermal treatment^23^,^28^. In addition, liquid-phase reduction of GO by hydrazine hydrate or NaBH_4_ is also involved in RGO modified g-C_3_N_4_ hybrid photocatalysts^23^. Since the reduction process depends on the reducing agent rather than CN material, the uncontrollable assembly will not give rise to an intimate interfacial contact between RGO and CN as required by efficient charge transportation. Therefore, it still remains a great challenge to develop an ideal CN/RGO heterostructure with extended region of light response as well as sufficient interfacial interaction that facilitates the application as visible light driven photocatalysis.

Herein, we demonstrated a two-step strategy for constructing high visible light active 2D/2D heterostructure by integrating a structure modification approach with a photo-assisted electrostatic assembly technique. The electronic structure of CN is modified by copolymerizing dicyandiamide precursor with an organic monomer aminobenzonitrile (X), so as to achieve the extended visible light absorption even after exfoliating to ultrathin nanosheets. Besides, the modification reversed the type of surface charge and thus enabled the modified CN nanosheets (CNX-NSs) to be readily assembled on the oppositely charged GO substrate during the following photo-assisted process. Only when the GO layer well-contacts with CNX-NSs, can it be reduced to RGO by photogenerated electrons. In this way, the 2D/2D CNX-NSs/RGO heterostructure directly formed in a uniform manner by π-π stacking and electrostatic attraction. The photocatalytic activity of the as-obtained hybrid was evaluated by methylene blue (MB) degradation. Based on characterizations and mechanism investigation, the remarkably enhanced visible photocatalytic efficiency is mainly ascribed to such delicately designed 2D/2D stacking heterostructure possessing improved visible-light utilization, electronic conductivity, and charge separation efficiency. This study demonstrates the possibility of constructing various multifunctional CN-based heterostrucutures with ideal optical/electrical properties and interfacial interaction towards diverse applications in environmental purification and energy conversion.

## Results and Discussion

### Synthesis strategy

**Figure 1:**
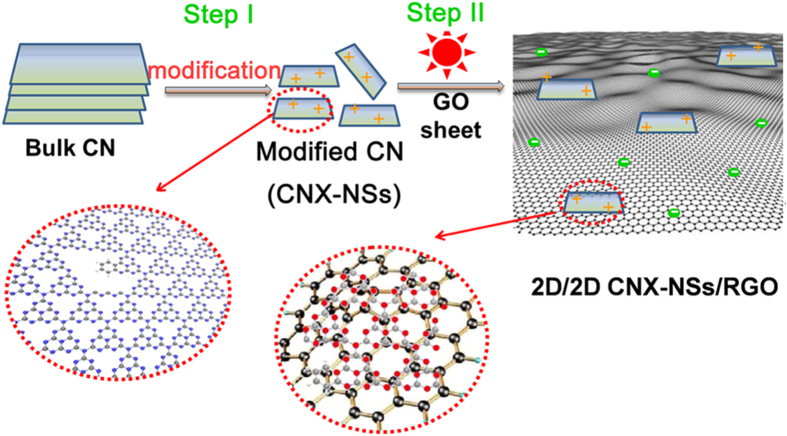
Schematic diagram for the synthesis of 2D/2D CNX-NSs/RGO hybrid. Step I: structural modification approach; Step II: photo-assisted electrostatic assembly technique.

The two-step synthetic procedure to construct well-contacted 2D/2D CNX-NSs/RGO heterostructure is illustrated in Fig. 1. At the first step, the modified CN nanosheets were obtained by copolymerization of dicyandiamide and aminobenzonitrile that followed by ultrasonication-assisted acid exfoliation procedure. The phenylene groups are incorporated into CN networks by copolymerization, achieving the extension of π-conjugated system and the remarkable red shift of optical absorption edge. Owing to the abundant -C-N- bonds in bulk CNX framework, the layered structure can be intercalated through H_2_SO_4_ protonation^11^. The intercalated layers will split into isolate layers due to the sonication and rapid release of heat when concentrated H_2_SO_4_ mixing with water. More importantly, the protonation brings forth the surface charge modification, i.e. changing from negative charged surface (−16.5 mV) to positive charged surface (+20.7 mV), as displayed in Figure S1. Interestingly, GO that synthesized by the modified Hummers’ method is found to exhibit a high negative charged surface (−42.6 mV) in neutral aqueous solution^29^,^30^,^31^. At the second step, the oppositely charged CNX-NSs will spontaneously assemble on GO layer by π-π stacking and electrostatic attraction via a photo-assisted method. Such interaction will benefit the formation of well-contacted interface and consequently facilitate the electron transportation during photoreduction reaction. On the other hand, the newly formed RGO layers function as ‘scaffolds’ to suppress the agglomeration of CNX-NSs. In this way, the exceptional 2D/2D CNX-NSs/RGO hybrid with an ideal heterostructure was fabricated.

#### Structural characterization

**Figure 2:**
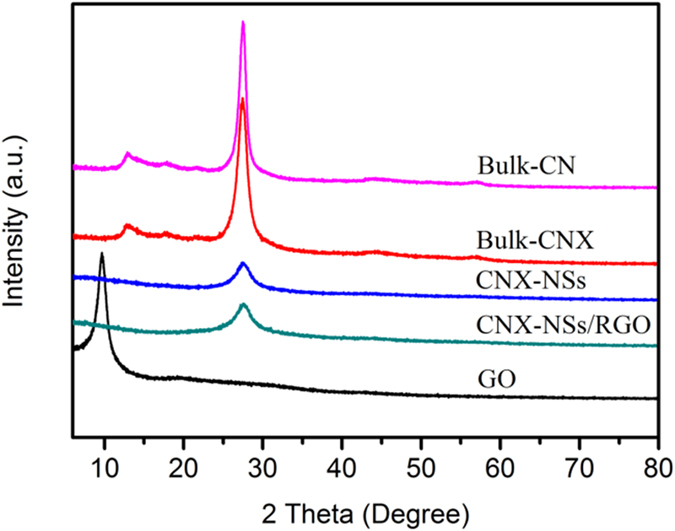
XRD patterns of the samples.

XRD patterns of as-prepared samples are displayed and compared in Fig. 2. For CN materials, two typical diffraction peaks at 2θ = 13.1° and 27.5° are attributed to the (100) in-plane repeat tri-s-triazine units and (002) interlayer stacking of graphite-like structures, respectively. The slight weakening of the characteristic peaks is observed in molecularly grafted product (CNX), suggesting the copolymerization blocking the in-plane periodic linkage as well as interlayer stacking by introducing phenylene groups into CN networks^14^,^18^. When exfoliating into ultrathin NSs, the interlayer stacking of CN is significantly weakened with a sharp decrease in the (002) diffraction of CNX-NSs, although some exfoliated layers are inclined to form agglomerates during the preparation of powders for XRD measurement^11^. Meanwhile, the disappearance of (100) in-plane diffraction of the CNX-NSs can be ascribed to the decreased planar size by ultrasonication-assisted acid exfoliation^32^,^33^. A strong diffraction peak of GO at 10.3° corresponds to the (001) diffraction peak of interlayer spacing^29^. For CNX-NSs/RGO hybrid, the same diffraction peak as CNX-NSs indicates that the basic chemical structure of C and N remained unchanged during GO reduction reaction. Meanwhile, the successful reduction of GO to RGO is confirmed because of the absence of GO characteristic peak. Notably, no diffraction peak of RGO is observed in the hybrid due to the low content and fairly weak diffraction intensity of RGO^34^.

**Figure 3:**
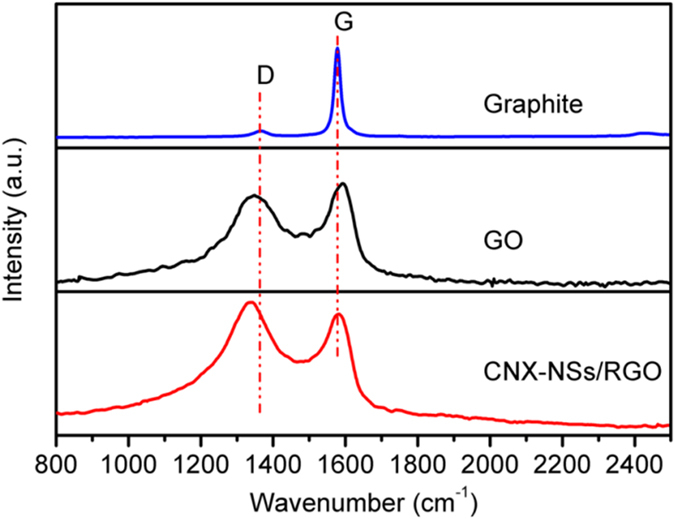
Raman spectra of graphite, GO, and CNX-NSs/RGO hybrid.

Raman spectroscopy as a powerful technique was used to further characterize the structure and electronic properties of carbon materials (Fig. 3). GO sample displays two distinct Raman response signals. The D band located at 1347 cm^−1^ corresponds to the internal structural defects, edge defects, and dangling bonds. The G band at 1592 cm^−1^ originates from the stretching mode of symmetric C–C bonds^35^. Compared to natural graphite, a significant increase in the D/G intensity ratio (*I*_D_*/I*_G_) from 0.11 to 0.87 is observed in GO, indicating the disruption of the symmetrical graphitic lattice. Meanwhile, both D and G peak obviously broadened because of the introduction of hydroxyl and epoxy groups by the modified Hummers’ method^34^. For CNX-NSs/RGO hybrid, D and G bands with much greater *I*_D_*/I*_G_ ratio (1.09) were observed, suggesting a more disordered structure due to the elimination of oxygen-containing groups by photogenerated electrons^36^. Moreover, the obvious shifts of the D and G bands in the hybrid arise from the interfacial interactions with CN^37^.

**Figure 4:**
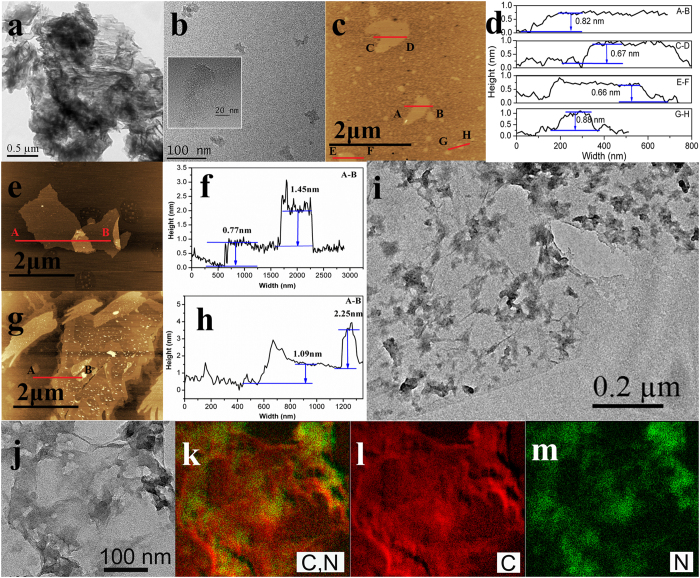
TEM images of (**a**) bulk CNX and (**b**) exfoliated CNX-NSs sol catalysts. The inserts in b is the magnified TEM image. AFM images and the corresponding cross-sectional analysis of (**c**,**d**) CNX-NSs sol, (**e**,**f**) GO, and (**g**,**h**) CNX-NSs/RGO. (**i**) TEM image of CNX-NSs/RGO hybrid. (**j**) The magnified TEM image and (k-m) the corresponding elemental mapping images of CNX-NSs/RGO hybrid.

The influence of exfoliation on the microscopic morphology was investigated by TEM and AFM observations. As shown in Fig. 4a, bulk CNX is composed of a number of micrometer sized layers. After ultrasonication-assisted acid exfoliation, the CNX-NSs in sol present an ultrathin layered structure without obvious bulk domains (Fig. 4b). The randomly selected nanosheets are measured by AFM, displaying a planar size of 50–100 nm and a thickness of 0.66–0.88 nm (about 2–3 layers) according to the theoretical thickness of ~0.33 nm (Fig. 4c,d)^11^. A line scan of the AFM height profile reveals the monolayered GO sheets with a topographic height of ~0.77 nm, consistent with the theoretically predicted value of ~0.8 nm (Fig. 4e,f)^38^. The thicker profile of hybrid (2.25 nm) indicates the successful assemble of CNX-NSs on the photoreduced RGO sheets by employing such a green photoreduction method (Fig. 4g,h). The TEM images in Fig. 4i,j and the corresponding elemental mapping images in Fig. 4k–m identified the small CNX nanosheets supporting on the semitransparent RGO layers. Both CNX-NSs and RGO well-maintained their original 2D structure to the formation of exceptional 2D/2D structure with well-contact interface, which is probably attributed to the π-π stacking and electrostatic attraction between oppositely charged partners^18^,^23^.

**Figure 5:**
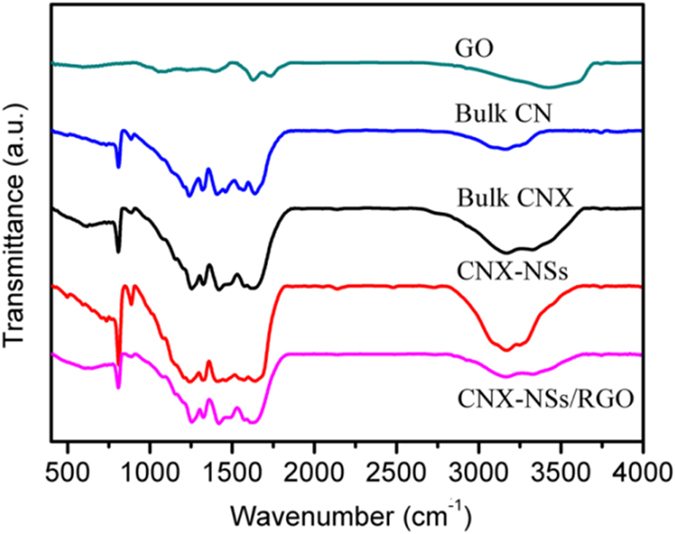
FT-IR spectra of the as-prepared samples.

The structural information of as-prepared samples was analyzed by FT-IR spectra, as displayed in Fig. 5. In the spectrum of GO sample, distinct peaks at 1065, 1258, 1390, 1625, 1730, and 3431 cm^−1^ can be assigned to C–O–C, phenolic C–OH, carboxyl C–O, aromatic C=C, C=O, and O–H stretching, respectively^24^. For CN sample, the peaks in the region from 1200 to 1700 cm^−1^ are attributed to the feature-distinctive stretching modes of aromatic CN heterocycles, arising from trigonal (N–(C)_3_) from full condensation and bridging C–NH–C from incomplete condensation^10^. The sharp peak at around 810 cm^−1^ corresponds to the breathing mode of the tri-s-triazine units. The broad peaks between 3000 and 3600 cm^−1^ are contributed by N–H and O–H stretching of free amino groups and adsorbed hydroxyl species^23^. FT-IR spectra of CNX, CNX-NSs and CNX-NSs/RGO all show almost identical peaks to pristine CN, indicating that the molecular grafting, exfoliation, and electrostatic assembly did not destroy the in-plane tri-s-triazine units. Besides, the diminishment of the characteristic peaks of GO verifies that most of the oxygen-containing groups in GO have been reduced by CNX-NSs during photoreduction process.

**Figure 6:**
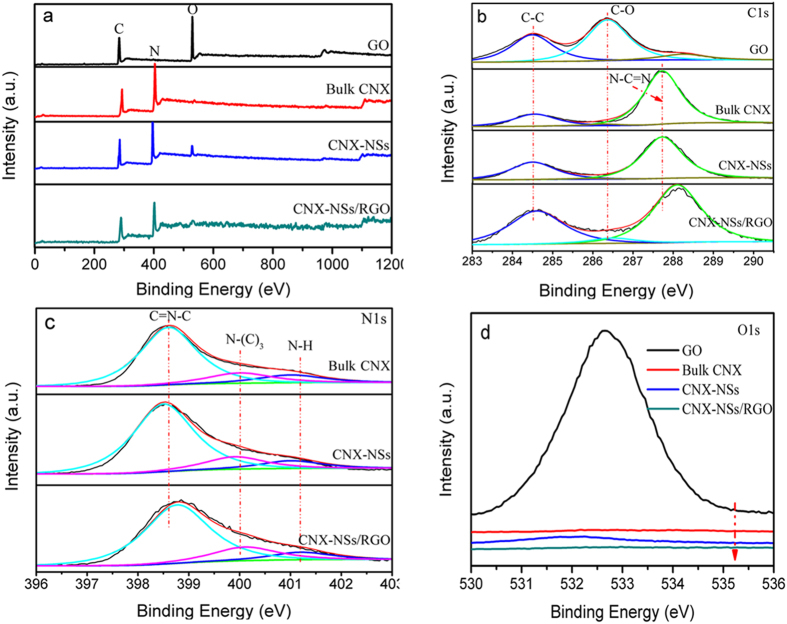
The XPS survey spectra (**a**), high-resolution C1s spectra (**b**) high-resolution N1s spectra (**c**), and high-resolution O1s spectra (**d**) of GO, bulk CNX, exfoliated CNX-NSs, and CNX-NSs/RGO hybrid.

The chemical states and compositions were investigated by XPS and elemental analysis. According to the survey spectra, all samples except GO contain only C, N and O elements (Fig. 6a). Since there is no obvious shift of the binding energy of C1s, N1s, and O1s core electrons in CNX and CNX-NSs (Fig. 6b–d), the local chemical environment of the elements are conserved after exfoliation. It is noted that the amount of O absorbed or bonded on nanosheets is higher than that on bulk material (Table S1), which reflects the oxidization of CN groups by acid exfoliation^18^. The high-resolution C 1 s spectrum of CNX displays two distinct peaks (Fig. 6b). The major peak at 287.8 eV is identified as sp^2^-hybridized carbon in aromatic N–C=N of CN network, and the weaker one at 284.6 eV is assigned to the adventitious standard reference carbon (graphitic carbon)^39^. Obviously, the intensity of C 1 s peak at 284.6 eV becomes much stronger in both GO and hybrid because of the overlap of C–C bonding configurations in adventitious reference carbon and GO (or RGO). For pure GO, there are two additional peaks located at 286.4 and 288.3 eV, corresponding to C–O in hydroxyl or epoxy groups, and HO–C=O bonds, respectively^40^. In the hybrid, the disappearance or dramatical diminishment of these peaks suggests the substantial removal of hydroxyl, epoxy, and carboxyl groups in GO. The O 1 s specta in Fig. 6d together with the surface element measurement in Table S1 also confirm the reduction of oxygen-containing functional groups. In comparison to CNX and CNX-NSs, the major peak located at 288.1 eV becomes broadening and can be deconvoluted into two peaks. The emergence peaks at 286.4 is due to the residual C–O bond after the reduction of GO. The higher shift (~0.3 eV) of the dominant peak (288.1 eV) of CN network reveals the existence of electronic interaction at CNX-NSs/RGO interface. The high-resolution N 1 s spectra can be deconvoluted into three peaks at 398.6, 399.9 and 401.2 eV (Fig. 6c), which are ascribed to sp^2^-hybridized aromatic N of the tri-s-triazine rings (C=N–C), bridging N in the form of N–(C)_3_, and amino groups (N–H) from incomplete condensation of dicyandiamide^41^. Similar to C 1 s, the binding energy of N 1 s in the hybrid shows ~0.2 eV higher than that in CNX or CNX-NSs, resulting from the interaction with RGO decreasing the electron intensity on the N atom^42^,^43^. Since adventitious graphitic carbon is introduced as the standard reference carbon, the atomic C/N ratio in CN-based materials cannot be accurately measured by XPS analysis. The elemental analysis is conducted to analyze the chemical composition, as summarized in Table S2. The atomic ratio of C/N is measured to be 0.66 in both CNX and CNX-NSs, suggesting the incomplete condensation of dicyandiamide. RGO incorporation increases the C/N ratio to 1.04, so the actual weight proportion of RGO can be deduced to be ~18.7%, close to the theoretical values of 20%.

#### Optical property

**Figure 7:**
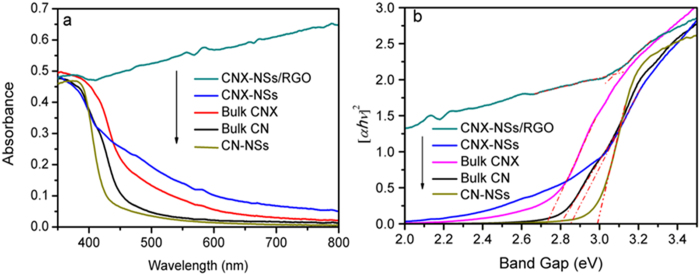
UV-vis DRS of the samples (**a**) and Tauc Plot of (*α*h*ν*)^2^ as a function of photon energy (h*ν*) (**b**).

The optical absorption properties play a critical role in determining the photocatalytic performance, especially for the visible-light-driven photocatalysis. The changes in UV-vis DRS as well as energy gap of CN network by molecular grafting, exfoliation, and coupling with RGO are recorded in Fig. 7. The pristine CN shows an absorption edge at ~443 nm^44^. The gap energy is estimated to be 2.80 eV according to the converted photon energy spectra in Tauc plot. Noticeably, the exfoliation process caused an obvious blue shift to 415 nm, accompanying by the bandgap broadening to 2.99 eV, which is consistent with literatures^9^,^10^,^11^. It was reported that the conduction band (CB) and valence band (VB) edges shifted in opposite direction after exfoliation due to the quantum effect^45^. Fortunately, the molecular grafting of remarkably altered the electronic structure so that the absorption edge onset of CNX is extended to ~600 nm^14^,^18^. Although the blue shift is unavoidable during exfoliation, the CNX-NSs still exhibit much wider utilizable light range than CN or CN-NSs. After coupling with RGO, the hybrid displays broad background absorption and improved light absorption intensity. The gap energy of the 2D/2D CNX-NSs/RGO hybrid is estimated to be 3.08 eV. As widely reported by literatures, GO coupling have little influence on the intrinsic energy levels or electron transitions of CNX-NSs^46^,^47^. The higher gap energy of hybrid than that of CNX-NSs powders implies that the exfoliated CNX nanosheets may maintain the original sheet-like structure in the presence of RGO rather than restack to bulk structure.

**Figure 8:**
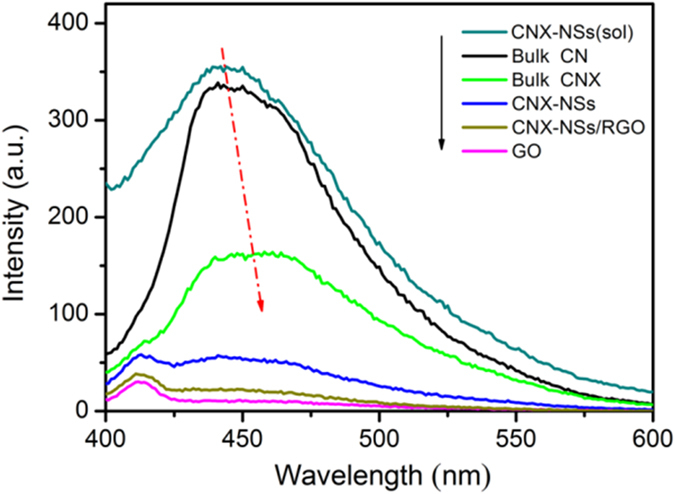
Photoluminescence spectra of the as-prepared samples under 360 nm excitation.

Further evidence can also be found in the PL spectra (Fig. 8). PL emission of the molecularly grafted CNX displays an obvious shift towards longer wavelength as compared with prisitine CN. More importantly, PL measurement can be employed to study the excited state and compare the photocatalytic efficiencies because PL emission arises from the recombination of photogenerated e^−^–h^+^ pairs. Pristine CN exhibits strong photoluminescence with an emission peak at ~440 nm that is equivalent to 2.8 eV. This strong band is attributed to the interband electronic transitions and excitonic PL phenomenon that caused by surface vacancies or defects^24^. An evident fluorescence quenching is observed for molecularly grafted sample CNX, indicating that the extended π-conjugation system with surface molecular heterojunction can effectively reduce the e^−^–h^+^ recombination rate^14^,^15^. Apparently, PL emission noticeably increased in the exfoliated CNX-NSs hydrosol^9^,^10^,^11^, implying that acid etching induced more surface vacancies or defects. After drying the hydrosol to powders, the isolate layers inclined to reassembling to bulk structure, accompanying with the self-repairing of surface vacancies and defects. As a result, the PL phenomenon dramatically decreased. In contrast, when the isolate CN layers assembled on RGO sheets, the extent of the FL quenching is found to be the greatest, indicating that RGO effectively decreased the recombination of photoinduced e^−^–h^+^ pairs of CN as an electron acceptor.

#### Evaluation of the photocatalytic performance

**Figure 9:**
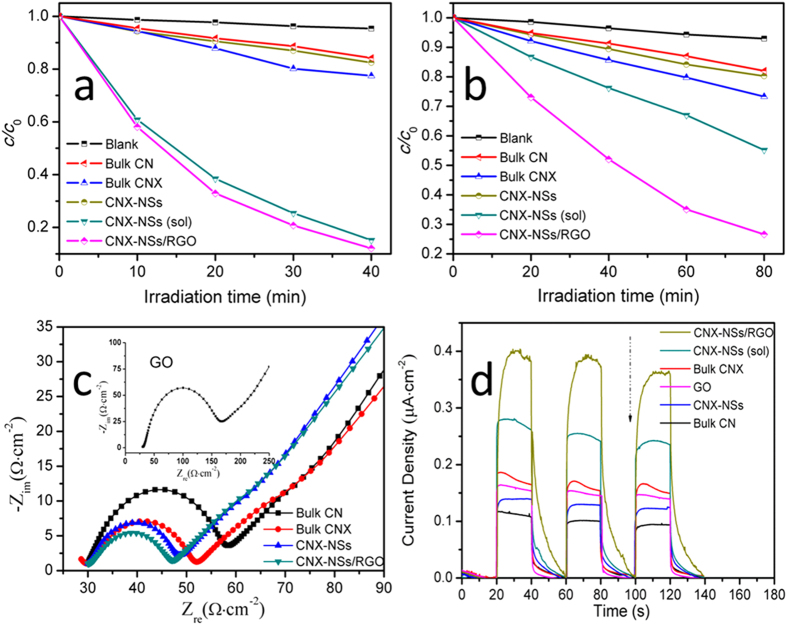
Photodegradation curve of MB dye over the prepared photocatalysts under full spectrum (**a**) and visible light (**b**) irradiation. (**c**) Nyquist plots of the as-prepared samples. (**d**) Photocurrent-time curves of as-prepared samples under visible light irradiation.

CN based photocatalysts have been inherently applied in environmental purification and energy conversion owning to its excellent visible light activity. The photocatalytic behaviors of as-prepared materials are evaluated and compared through MB degradation under full spectrum and visible light (λ ≥ 420 nm) (Fig. 9a,b). The corresponding rate constants are calculated from Figure S2 and compared in Table S3. Before light illumination, 26 mg L^−1^ MB suspensions containing photocatalysts are stirred for 30 min in the dark to attain adsorption-desorption equilibrium (Figure S3). Similar to other RGO-based systems, the highest absorption ability is observed in hybrid system, largely assigning to the noncovalent adsorption by π-π stacking between the aromatic regions of dye and catalysts^48^. In the blank experiment, nearly no degradation of MB by direct photolysis is observed in the absence of photocatalyst. As expected, the molecularly grafted sample CNX exhibits higher photoactivity towards MB degradation than pristine CN under both full spectrum and visible light, implying the decreased e^−^–h^+^ recombination as well as enhanced visible light absorption capability by phenylene incorporation. The improved charge carrier separation can also be confirmed by EIS and photocurrent measurements (Fig. 9c,d). The semicircle at high frequencies in the Nyquist diagrams is in accordance with that in the electron-transfer-limited process and the semicircle diameter represents the electron-transfer resistance (*R*_et_) across the electrode/electrolyte. The lower semicircle diameter in CNX than that in pristine CN indicates the higher electron transfer efficiency in the non-photoexcited state. As further evidence, CNX possesses higher intensity of photocurrent signal than pristine CN, reflecting the improvement of photoinduced charge carriers transfer and separation by molecular grafting. In comparison to bulk CNX, the exfoliated CNX-NSs hydrosol exhibits significantly enhanced photoactivity under full spectrum but a little enhancement under visible light. The highest performance under full spectrum demonstrates the synergistic effect of molecular grafting and exfoliation on charge carriers transfer and separation, i.e. molecular grafting extended π-conjugation system of CN network and created surface molecular heterojunction; exfoliation decreased the size of 3D bulk material in one dimension. Under visible light irradiation, the enhancement in charge carriers separation by exfoliation are partly offset by the blue shift of absorption edge. However, it will be difficult for hydrosol photocatalyst to be recycled from economic consideration. The photocatalytic efficiency of CNX-NSs powders should be taken into account. Since the exfoliated CNX layers reassembled into bulk structure during drying into powders, the photoactivity as well as photocurrent response dramatically decreased. This result implies the isolated CNX layers can only maintain their original morphology in aqueous solution.

Surprisingly, the photocatalytic activity significantly enhanced under full spectrum and visible light when assembling exfoliated CNX layers on RGO sheets (Fig. 9a,b). The hybrid exhibits 88% and 73% MB degradation under full spectrum for 40 min and visible light for 80 min, which is 7.7- and 5.1-folds enhancement over CNX catalysts (or 12.5- and 7.0-folds over CN), respectively. In the presence of RGO, the well-contacted 2D/2D stacking morphology is formed by π-π stacking and electrostatic attraction between oppositely charged CNX-NSs and RGO with comparable π-conjugated structure, inhibiting the reassembling of exfoliated CNX layers. Nyquist plot of the hybrid displays the highest electronic conductivity, suggesting that GO is reduced to RGO and benefits the electron transport (Fig. 9c). Due to the superior electron transport property of RGO, the photoinduced electrons could rapidly transfer from the CB of semiconductors to RGO through interfacial interactions^48^. In this way, RGO functions as a competitive acceptor material to suppress the recombination of charge carriers on CNX-NSs, leading to higher photocurrent response (Fig. 9d). Therefore, the excellent photocatalytic efficiency of CNX-NSs/RGO hybrid can be attributed to the improved charge carriers separation and visible light absorption that achieved by such a delicately designed two-step strategy, i.e. structural modification approach and photo-assisted electrostatic assembly technique.

As a competitive electron acceptor, RGO has been reported to possess the concentration-dependent influences on the photocatalytic performance of semiconductors^23^,^24^,^25^,^26^,^27^,^47^. All of the CNX-NSs/RGO hybrids exhibit better photoactivity than CNX-NSs powders, suggesting that RGO sheets function as scaffolds to suppress the aggregation of exfoliated CNX-NSs layers and as electron collector to facilitate the charge carriers separation (Figure S4). The lower RGO content in the heterostructure cannot provide adequate intimate contact area to suppress the aggregation of exfoliated CNX-NSs layers as well as the recombination of charge carriers. When the RGO content reaches 20%, a further increase will be unfavorable for improving the photocatalytic activity of CNX-NSs because excess RGO will shield some of light arriving at the surface of CNX-NSs and then cause a decrease in degradation rate. Meanwhile, large amount of RGO might occupy active sites on the CNX-NSs surface and block subsequent reactions with dye molecules. Therefore, an appropriate RGO ratio in heterostructure is of great significance for the interfacial interaction with CNX-NSs for improving the photocatalytic performance.

The stability of CNX-NSs/RGO hybrid is investigated by measuring the cyclic photocatalytic efficiency towards MB degradation through consecutive five cycles. After every test cycle, the photocatalyst is centrifuged, washed, and dried. As shown in Figure S5, the photocatalytic activity does not exhibit any significant loss after five cycles, suggesting the high stability against photocorrosion. The FT-IR spectra of hybrid photocatalyst before and after photocatalytic reaction are recorded in Figure S6 to confirm the stability of photocatalysts. The spectrum of used CNX-NSs/RGO catalyst does not show any difference from fresh one, further demonstrating the stable chemical skeleton of CNX-NSs and RGO throughout whole process.

### Mechanisms of the enhanced photocatalytic performance of CNX-NSs/RGO hybrid

**Figure 10:**
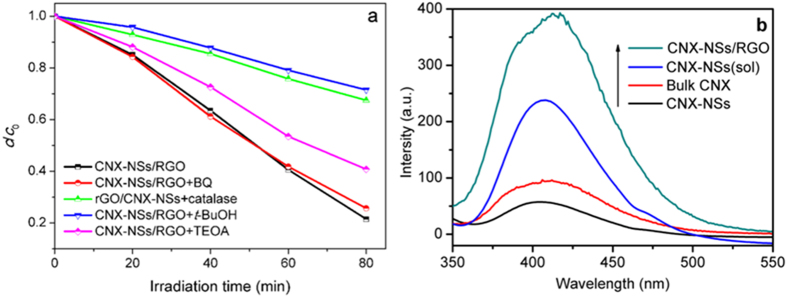
Trapping test of photogenerated holes and radicals in CNX-NSs/RGO photodegradation system under visible-light irradiation. (**b**) PL spectra for detection of H_2_O_2_ generation over different photocatalysts under 30 min visible light irradiation. The excitation wavelength is set as 290 nm.

Although the role of RGO on the photocatalytic performance of CNX-NSs is well-known as inhibitors for photoinduced carriers recombination, the contributions of active species that produced by charge carriers to the degradation of organics are unclear so far. Investigation of the exact influence of RGO coupling would be useful for an indepth study of the photocatalytic mechanism in CNX-NSs/carbon composites and for the development of highly active photocatalysts. Detailed discussions for the photocatalytic mechanism of RGO/TiO_2_ hybrid are presented in our previous work, elucidating that the enhancement mainly came from holes left in the TiO_2_ crystals rather than electrons transferring to RGO^34^. The dominant role of holes was also observed in other RGO coupling inorganic semiconductor systems^49^,^50^. Different from TiO_2_ photocatalysts, the VB position of CNX-NSs is reported to be ~1.47 eV (vs. SHE), more negative than that of TiO_2_ (2.7 vs. SHE) and ^·^OH/OH^−^ (1.99 eV vs. SHE), indicating the relatively weak oxidation capability of holes in CNX-NSs. To evaluate the contribution of these active species, targeted scavengers were introduced into the photodegradation system under visible light. The trapping experiments are conducted by adding benzoquinone (BQ) for O_2_^−·^, catalase for H_2_O_2_, tert-butanol (*t*-BuOH) for ^·^OH, and triethanolamine (TEOA) for h^+^ (Fig. 10a). Apparently, the photodegradation rate is somewhat reduced with the addition of TEOA, revealing the less dominant role of h^+^ in CNX-NSs/RGO hybrid than that in RGO/TiO_2_ systems. For reactive oxygen species (ROS), the addition of BQ caused a slight decrease in photocatalytic activity, indicating the least importance of O_2_^−·^ species. On the contrary, catalase and *t*-BuOH significantly inhibited MB degradation, suggesting that the H_2_O_2_ and ^·^OH are critically important in the photocatalytic process. The photoinduced e^−^ can be captured by molecular O_2_ to generate ROS and initiate the degradation process (eqs 1, 2, 3, 4, 5).


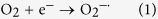



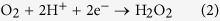



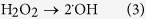



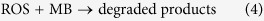



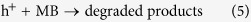


**Figure 11:**
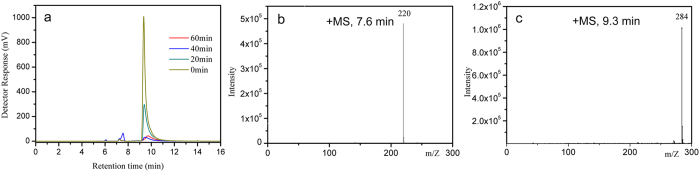
(**a**) LC data of photodegraded MB at given time intervals. (**b**,**c**) MS signals of the major detected intermediates eluting at 7.6 and 9.3 min.

**Figure 12:**
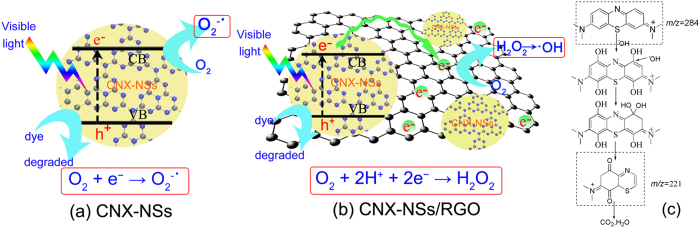
Comparison of possible photocatalytic mechanism of CNX-NSs (**a**) and the well-contacted 2D/2D CNX-NSs/RGO heterostructure (**b**) under visible light irradiation. (**c**) Possible degradation pathway from MB dye during photocatalytic reaction^51^.

The well-contacted RGO acted as an electron reservoir and transferred abundant photogenerated electrons to O_2_, favoring a two-electron process to H_2_O_2_^45^. It was found that the degradation rate in the presence of catalase was similar to that of *t*-BuOH. Since holes are incapable of oxidizing adsorbed OH^−^ groups into ^·^OH, the generation of ^·^OH mainly comes from the decomposition of H_2_O_2_. The amount of H_2_O_2_ produced on the photocatalysts under visible light irradiation was compared by using a selective fluorescent probe (Fig. 10b and S7). The amount of H_2_O_2_ gradually increased with the irradiation time. More H_2_O_2_ was detected over CNX-NSs/RGO hybrid, indicating that RGO coupling greatly improved the charge separation efficiency and accelerated the visible photocatalytic performance of CNX-NSs. To provide more information for MB degradation process, the reactions at given time intervals were monitored by LC-MS. As displayed in Fig. 11, the intensity of MB peak (*m*/*z* = 284) steeply reduced with irradiation time, which confirmed the MB degradation during photocatalytic process. The MS signal relative to LC peak at 6.1 min is too low to be identified from the MS background. After 40 min irradiation, the intensity of LC peak at 7.4 min dramatically increased and then decreased with prolonged irradiation time, reflecting the formation of intermediate product and the subsequent degradation. The LC peak at 7.4 min corresponds to a fragment with *m*/*z* = 220, which can be ascribed to the cleavage of one aromatic ring after OH group substitution. This intermediate product has also been discovered in the photo-Fenton reaction, suggesting the crucial role of ^·^OH in MB oxidation by such a CNX-NSs/RGO hybrid system^51^. On the basis of the above discussion, a visible photocatalytic mechanism over 2D/2D CNX-NSs/RGO hybrid is depicted in Fig. 12.

## Conclusions

An efficient metal-free visible photocatalyst is successfully developed by assembling molecularly grafted CN nanosheets on RGO layers via a two-step strategy, which consists of structure modification and photo-assisted electrostatic assembly processes. Compared to pristine bulk CN, the molecularly grafted CNX-NSs obtained from the first step possess improved visible light harvesting, charge separation efficiency, and water dispersibility. In the second step, GO was reduced to RGO by capturing the photogenerated electrons from CNX-NSs. Simultaneously, the oppositely charged CNX-NSs were assembled on the surface of RGO layers via π-π stacking and electrostatic attraction. Detailed characterizations verified the reduction of GO and the formation of well-contacted 2D/2D CNX-NSs/RGO heterostructure. Further, XPS analysis demonstrates the strong interaction between them. The investigation in optical property indicates that RGO coupling inhibits the restacking of the exfoliated CNX layers when drying into powders.

As a result, the hybrid exhibits remarkable 12.5- and 7.0-folds enhancement of MB degradation over pristine CN catalysts under full spectrum and visible light irradiation, respectively. More importantly, the photocatalytic efficiency of hybrid achieves 1.1- and 2.4-times that of CNX-NSs sol catalyst under full spectrum and visible light irradiation, respectively. Through trapping experiments and H_2_O_2_ detection, it was further shown that the enhancement mainly came from electrons transferring to RGO to generate more H_2_O_2_ via a two-electron process. Therefore, this significantly enhanced photocatalytic activity over hybrid catalyst can be attributed to the improved light utilization, high electronic conductivity, and superior charge separation efficiency by rational design of well-contacted 2D/2D CNX-NSs/RGO heterostructure. Overall, this work not only yields high efficient metal-free visible photocatalysts but also provides deeper insight into the enhanced mechanisms of RGO coupling CN heterostructures. It is expected that such photo-assisted electrostatic assembly strategy could be extended to the development of various multifunctional CN-based heterostructures towards diverse applications in environmental purification and energy conversion.

## Experimenal

### Materials

Graphite powder was obtained from Alpha Aesar. Dicyandiamide, sulfuric acid (H_2_SO_4_), sodium nitrate (NaNO_3_), potassium permanganate (KMnO_4_), 30% hydrogen peroxide and other materials were purchased from Shanghai Pure Chemical Co., Ltd, China.

### Preparation of CNX-NSs and GO

GO was prepared by oxidation of the natural graphite using a modified Hummers’ method^34^. Typically, natural graphite was reacted with concentrated H_2_SO_4_, NaNO_3_, and KMnO_4_. Then H_2_O_2_ was added to the mixture after the reaction completed. The product was washed and centrifuged using dilute HCl and deionized water for several times. Finally, the precipitate was dried and dispersed in deionized water with a mass concentration of GO of 4.94 mg mL^−1^ by ultrasonication for further use. Molecularly grafted CN nanosheets (CNX-NSs) were prepared by ultrasonication-assisted acid exfoliation of CNX. 3 g of dicyandiamide and 0.05 g of 2-aminobenzonitrile were mixed in 15 mL of water under stirring at 373 K to remove the solvent. The resultant solid was dried under vacuum oven, milled into ultrafine podwers, and calcined at 550 °C for 4 h in air. The obtained sample was molecularly grafted bulk CN, labeled as CNX. The contrast sample CN was synthesized in the same method without adding 2-aminobenzonitrile. Then, the bulk materials were exfoliated to ultrathin nanosheets via ultrasonication-assisted acid exfoliation method. 300 mg of bulk CNX powders were milled well and then dispersed in 30 mL of concentrate sulfuric acid under stirring for 12 h at room temperature. The resulted aqueous suspension was diluted to 100 mL by deionized water and then treated by ultrasonication for 2 h. The mixture was centrifuged and washed with deionized water until its pH reached 7.0. The resulted precipitate was diluted to 30 mL by deionized water and then treated by ultrasonication for 2 h. Finally, the CNX-NSs colloid was separated from the residual unexfoliated bulk materials by centrifugating at 8000 rpm for 15 min. The mass concentration of CNX-NSs is calculated to be 0.7~1.2 mg mL^−1^ from several repeated experiments.

### Preparation of CNX-NSs/RGO hybrid

A stoichiometric ratio of GO and CNX-NSs colloids were dispersed in the mixture of 80 mL deionized water and 20 mL ethanol. The obtained suspension was sonicated by 1 h to disperse uniformly. Before irradiation, the suspension was degassed under N_2_ flow for 30 min. Then the photo-assisted electrostatic assembly was performed using mercury lamp as a light source under N_2_ flow for 6 hours. The light brown homogeneous solution then changed to suspensions with obvious dark sedimentations because of the hydrophobic property of RGO. As a result of electrostatic attraction, CNX-NSs preferred to nucleate at defects of RGO and separate from the reaction system. The obtained mixture was concentrated by rotary evaporation and then stirred at 70 °C to evaporate the residual solvent. The final CNX-NSs/RGO product was collected by vacuum drying at 60 °C and milled into ultrafine powders. According to the mass proportion of GO to CNX-NSs, the products were denoted as CNX-NSs/RGO-x%. The CNX-NSs/RGO hybrid that frequently described in this work has the default value of 20%.

### Characterizations

Zeta potentials were obtained from phase analysis light-scattering measurements performed on a Malvern Instruments Zetasizer Nano ZS90. Powder X-ray diffraction (XRD) data were recorded by a X’ Pert PRO instrument using Cu Kα irradiation (k = 1.5406 Å). The atom force microscopy (AFM) images were obtained on SPM9700. Raman spectra of the samples were obtained using Jobin Yvon LabRAM HR 800 equipped with a 532 nm laser (Raman microscope). The morphology was tested using SEM (Nova NanoSEM 450) and TEM Tecnai G2 F30 (FEI, Holland). Fourier transform infrared (FTIR) spectroscopy analysis was performed using FT-IR spectrophotometer (Perkin Elmer spectrum 1000) with KBr as the reference sample. Chemical compositions and binding energy of the samples were analyzed by X-ray photoelectron spectroscopy (XPS, AXIS-ULTRA DLD-600W) with C1s peak (284.6 eV) as a reference. Elemental analysis was performed with a Vario El element analyzer (Elementar Analysensysteme GmbH). UV-vis diffuse reflectance spectra (DRS) of the samples were recorded on a Varian Cary 5E spectrophotometer. Photoluminescence (PL) spectra of the samples were obtained using a fluorescence spectrometer (Hitachi F-7000). Intermediate compounds were identified by the liquid chromatography mass spectrometry (LC-MS, Agilent 1100 LC /MSD Trap). A reverse phase C18 column (5 μm particle size, 4.6 by 300 mm) was used. The flow rate was set at 0.33 ml min^−1^ and the injection volume was 100 μL. Before the analysis, all the solution was filtered with 0.2 μm filter. The mobile phase was a mixture of 40% actonitrile (with 0.1% formic acid) and 60% water (with 0.1% formic acid). The mass spectrometer conditions were as follows: capillary voltage, 3.0 kV; cone voltage, 60 eV; source temperature, 125 °C; desolvation temperature, 350 °C; desolvation gas flow, 250 L h^−1^.

### Photocatalytic activity evaluation

The photocatalytic activities of photocatalysts were measured by degrading aqueous solution of methylene blue (MB) under full spectrum and visible light irradiation. 5.0 mg of photocatalyst was suspended in 10 mL of an aqueous solution containing 26 mg L^−1^ MB. A 300 W Xe lamp was used as the light source. For visible light photocatalysis, a cutoff filter was settled to remove any radiation below 420 nm. Before irradiation, the suspensions were stirred for 40 min in the dark to establish an adsorption-desorption equilibrium. The adsorption capacities of the photocatalysts were tested by determining the MB remaining after stirring in the dark. At given time intervals, the suspension was sampled and centrifuged at 12000 rpm for 5 min. The changes in maximum absorption of MB absorption spectra versus irradiation time (*c*/*c*_0_ versus t) were obtained, which reflected the decrease in MB concentration.

### Electrochemical and photoelectrochemical measurements

Electrochemical impedance spectroscopy (EIS) and photocurrent was measured with a CHI660C electrochemical workstation (Chenhua Instruments, Shanghai, China) in a standard three-electrode system, which employed a platinum wire as the counter electrode and Ag/AgCl electrode as the reference electrode. 2 mg of powder was dispersed ultrasonically in 1 mL of water, and 75 μL of the resulting colloidal dispersion was drop-cast onto a piece of 10 mm × 10 mm indium-tin oxide (ITO) glass electrode. The electrode was dried in air at room temperature to eliminate water and heated at 60 °C to form modified ITO electrode. All investigated working electrodes were of similar thickness. 0.1 M Na_2_SO_4_ was used as the electrolyte for measurements.

### Trapping tests and H_2_O_2_ detection

To investigate the role of active species generated in the photocatalytic process, the trapping experiments were conducted by adding scavengers of free radicals and holes, benzoquinone (BQ) for O_2_^−·^, catalase for H_2_O_2_, tert-butanol (*t*-BuOH) for ^·^OH, and triethanolamine (TEOA) for h^+^ capture^34^. The analysis procedure was exactly identical to the above photocatalytic process. Various scavengers were added to the MB solution prior to addition of the photocatalyst. The adding amount of scavenger is 2 mM L^−1^. H_2_O_2_ generation was determined by a fluorescence assay. The p-hydrophenylacetic acid (POPHA) was oxidized to a fluorescence product 5,5′-dicarboxymethyl-2,2-dihydroxybiphenyl by a peroxidase-catalyzed reaction. 0.5 mL of catalyst solution was kept in dark for 40 min and then mixed with 0.5 mL of Tris buffer solution (0.1 M, pH 8.8) containing POPHA (8 mg) and horseradish peroxidase (2 mg). After reacting for 30 min, the suspension was centrifuged and the supernatant was sampled for fluorescence analysis at given time intervals.

## Additional Information

**How to cite this article**: Chen, J. *et al*. Toward High Performance 2D/2D Hybrid Photocatalyst by Electrostatic Assembly of Rationally Modified Carbon Nitride on Reduced Graphene Oxide. *Sci. Rep.* **6**, 37318; doi: 10.1038/srep37318 (2016).

**Publisher’s note**: Springer Nature remains neutral with regard to jurisdictional claims in published maps and institutional affiliations.

## Supplementary Material

Supplementary Information
