# Supplementary material for: Toward High Performance 2D/2D Hybrid Photocatalyst by Electrostatic Assembly of Rationally Modified Carbon Nitride on Reduced Graphene Oxide
Source: Sci Rep. 2016 Nov 17;6:37318. doi: 10.1038/srep37318 (PMC5112518; doi:10.1038/srep37318)
Supplement: Supplementary Information [file srep37318-s1.doc]

Supporting Information for

**Toward High Performance 2D/2D Hybrid Photocatalyst by Electrostatic Assembly of Rationally Modified Carbon Nitride on Reduced Graphene Oxide**

**Jian Chen, Xiaochan Xu, Tao Li,* Kannusamy Pandiselvi and Jingyu Wang***

School of Chemistry and Chemical Engineering, Key Laboratory of Material Chemistry for Energy Conversion and Storage (Ministry of Education), Huazhong University of Science and Technology, Wuhan, 430074, China

* E-mail: jingyu.wang@163.com (J. Wang); taoli@mail.hust.edu.cn (T. Li)


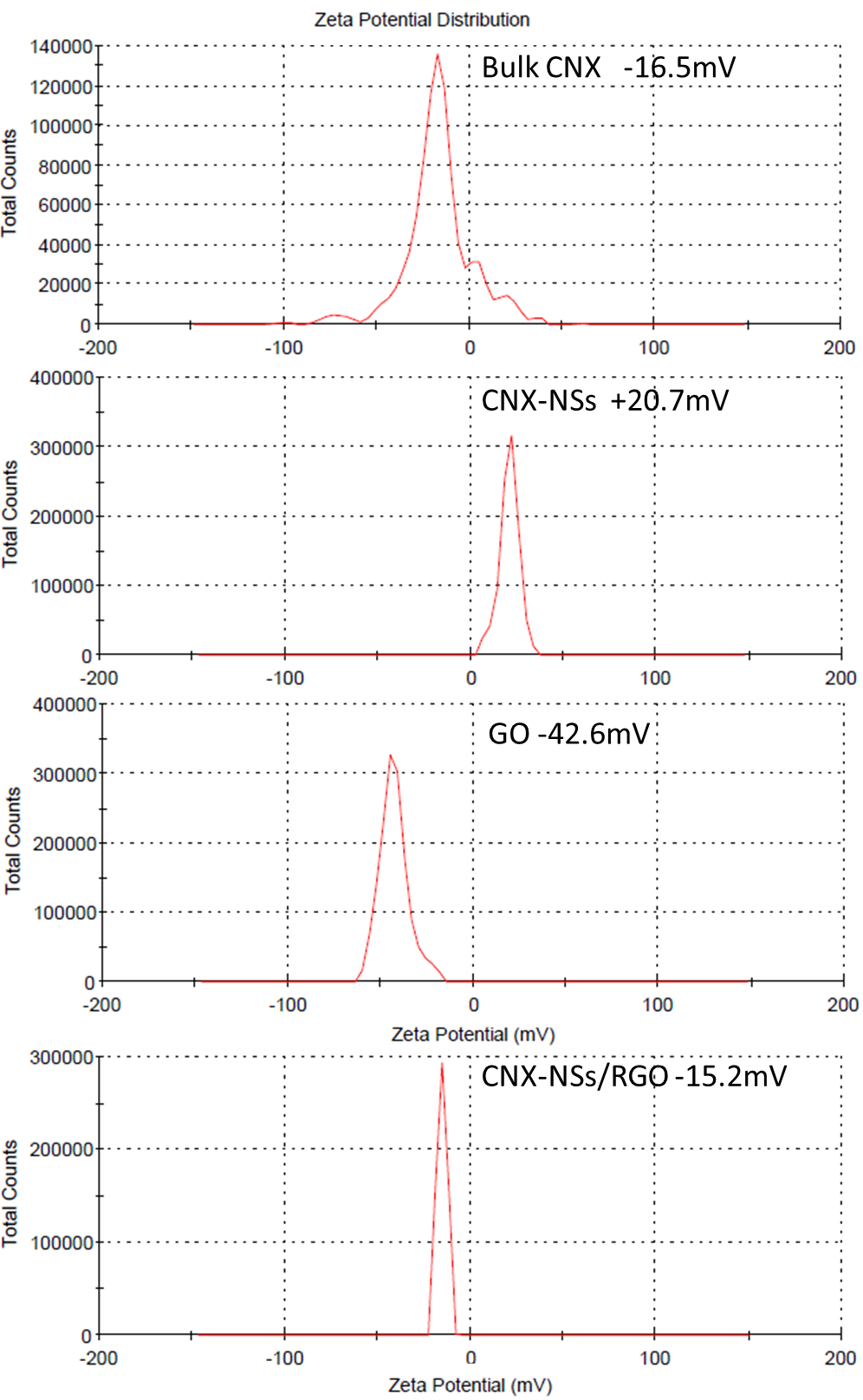


**Figure S1**. Zeta potentials of bulk CNX, CNX nanosheets (CNX-NSs), GO, and CNX-NSs/RGO hybrid.


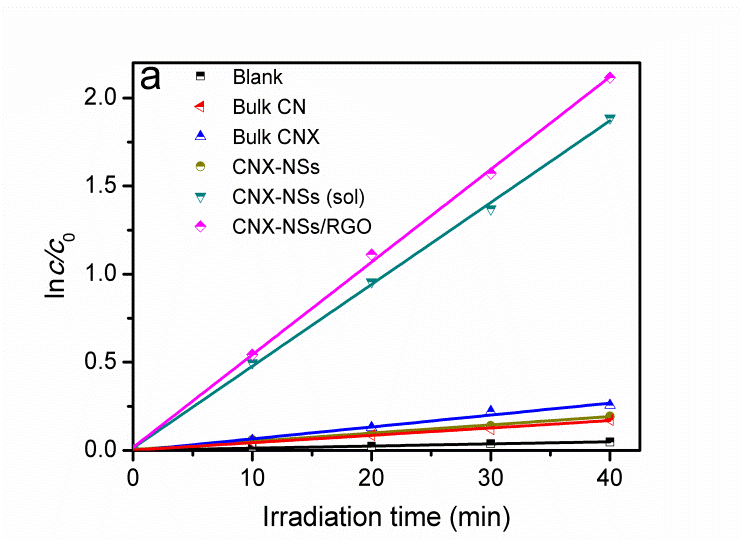

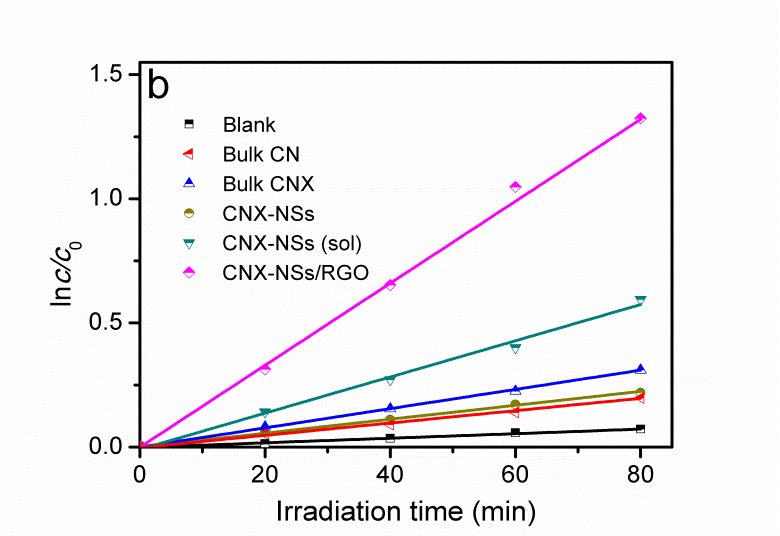


**Figure S2**. The first-order plots of MB concentration over the prepared photocatalysts according to the photodegradation curve in Figure 8a-b under full spectrum (a) and visible light irradiation (b).


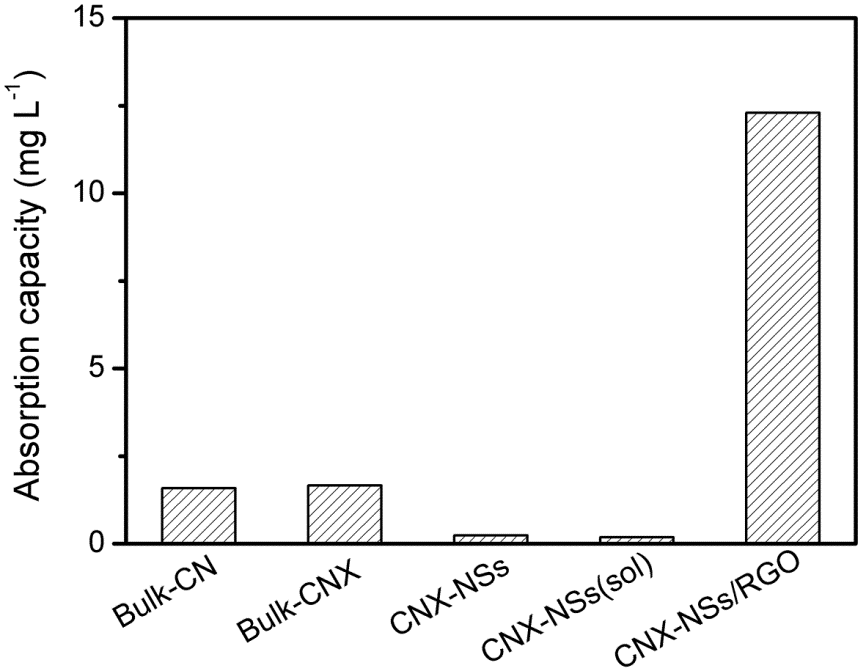


**Figure S3**. Bar plot showing the absorption capacity of MB dye after continuous stirring for 40 min in the dark.


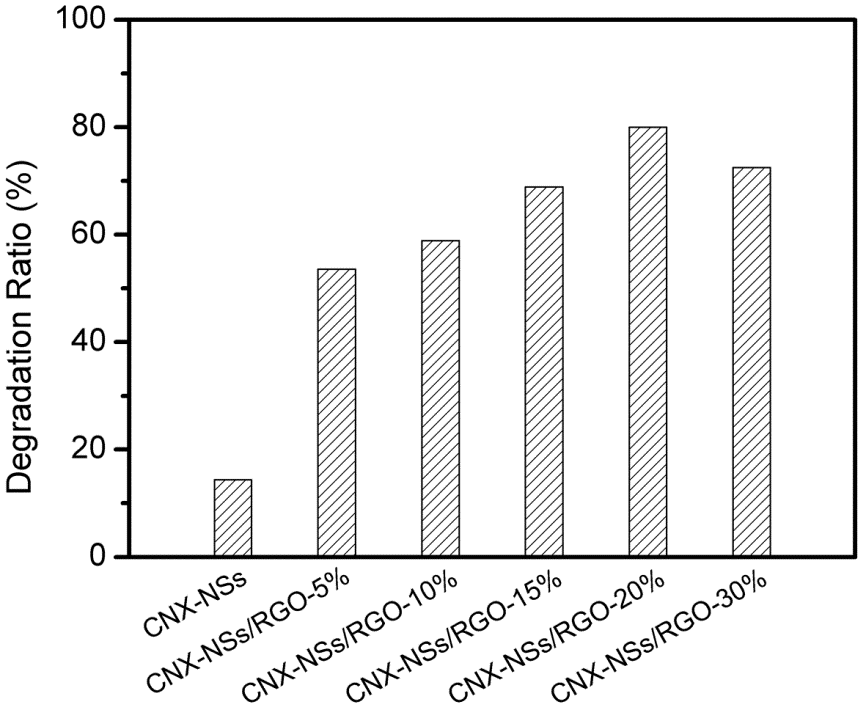


**Figure S4**. The photocatalytic performance of the CNX-NSs/RGO hybrids with different RGO content towards MB degradation under 30 min full spectrum irradiation.


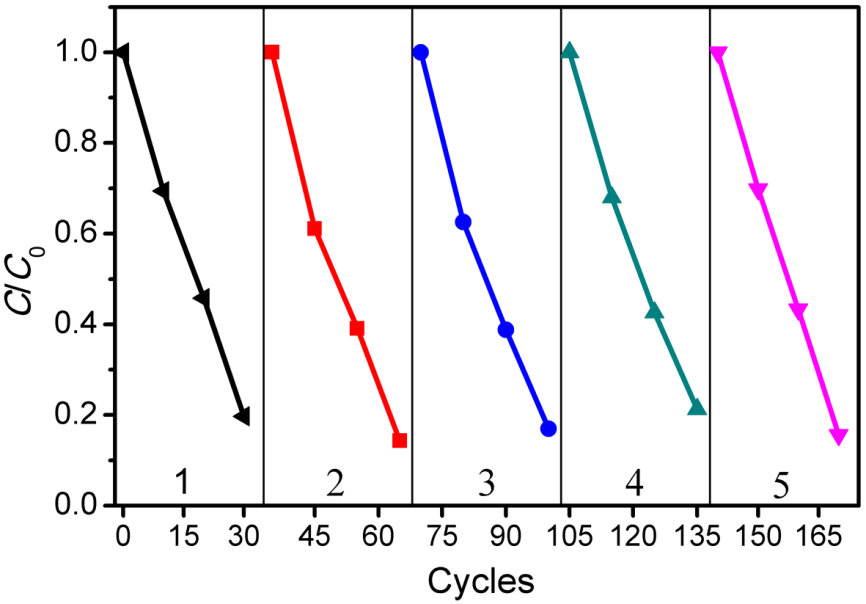


**Figure S5**. Cycle photodegradation of MB dye with CNX-NSs/RGO hybrid under full spectrum irradiation.


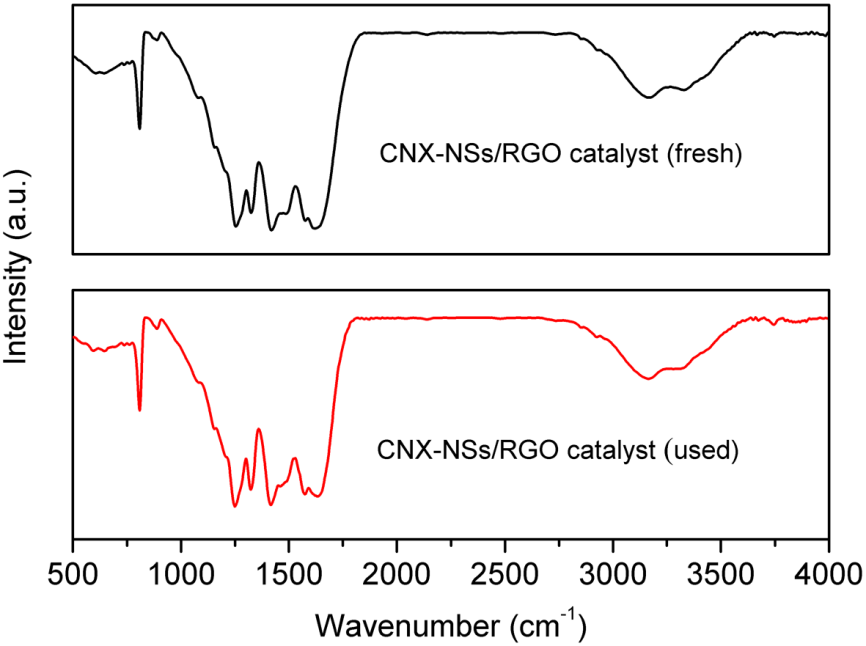


**Figure S6**. FT-IR spectra of the fresh and used CNX-NSs/RGO catalysts.


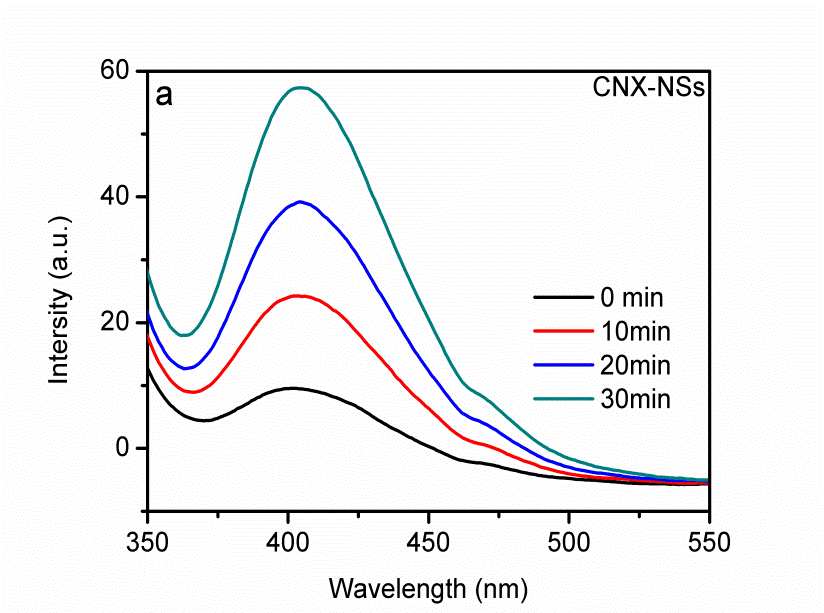

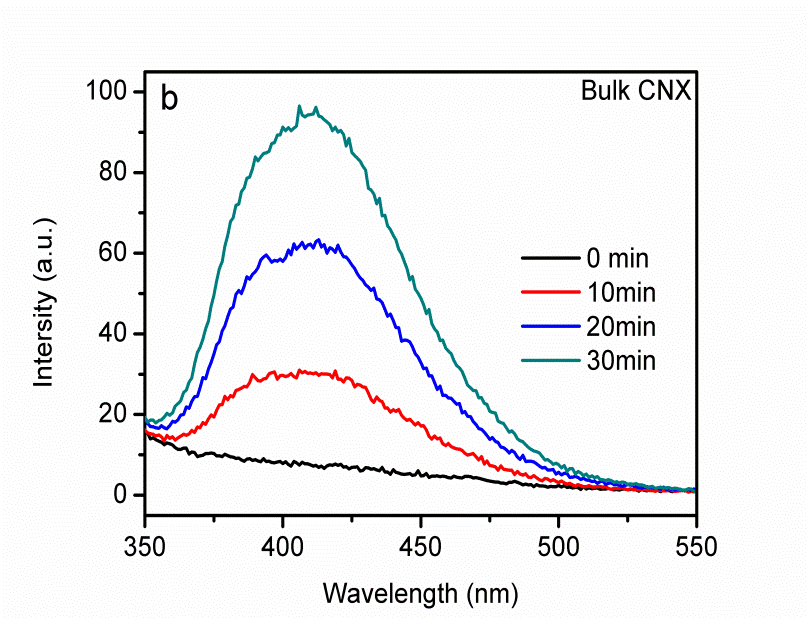


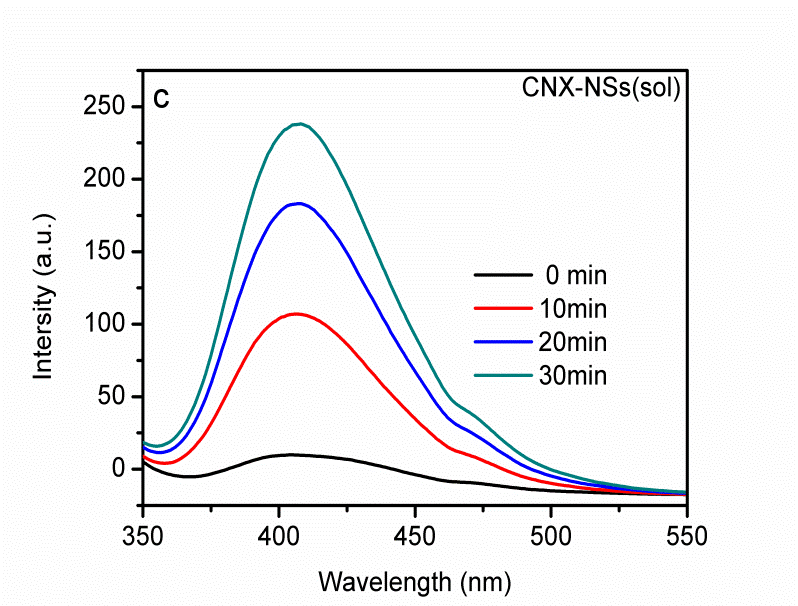

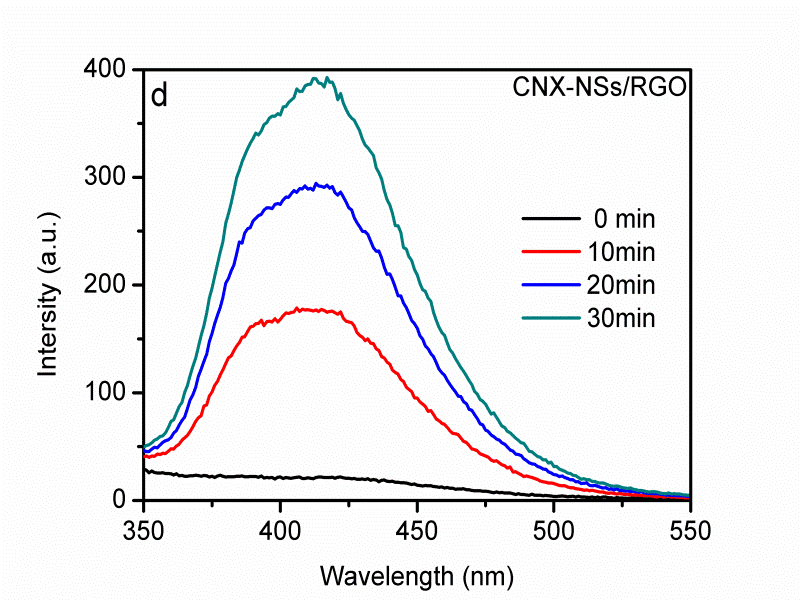


**Figure S7**. The amount of H_2_O_2_ produced on the photocatalysts under visible light irradiation.

Table S1. Chemical composition of the surface of the photocatalyst calculated from XPS.

| Photocatalyst | Atomic proportion (%) | | | |
| --- | --- | --- | --- | --- |
|  | C1s | N1s | O1s |  |
| Bulk-CN | 54.69 | 45.18 | 0.13 |  |
| Bulk-CNX | 42.90 | 55.55 | 1.55 |  |
| CNX-NSs | 43.86 | 50.44 | 5.70 |  |
| GO | 65.50 | - | 34.50 |  |
| CNX-NSs/RGO | 49.28 | 48.86 | 1.86 |  |

**Table S2**. Elemental analysis of the bulk CNX, CNX-NSs, and CNX-NSs/RGO hybrid. The reported values and their uncertainties are the average deviation of three replicates.

| Sample | bulk CNX | CNX-NSs | CNX-NSs/RGO |
| --- | --- | --- | --- |
| molar ratio of C:N | 0.66±0.01 | 0.66±0.01 | 1.04±0.01 |

**Table S3**. Degradation rate constants of MB dye over different photocatalysts.

| Samples | full spectrum irradiation | | visible light irradiation | |
| --- | --- | --- | --- | --- |
|  | *R*^2^ value | calculated values of  *k* (min^-1^) | *R*^2^ value | calculated values of  *k* (min^-1^) |
| Blank | 0.9936 | 0.0012 | 0.9915 | 0.0009 |
| Bulk CN | 0.9942 | 0.0042 | 0.9936 | 0.0024 |
| Bulk CNX | 0.9911 | 0.0068 | 0.9987 | 0.0033 |
| CNX-NSs | 0.9986 | 0.0047 | 0.9912 | 0.0028 |
| CNX-NSs (sol) | 0.9987 | 0.0465 | 0.9969 | 0.0072 |
| CNX-NSs/RGO | 0.9928 | 0.0526 | 0.9983 | 0.0169 |
